# TAK1 signaling regulates p53 through a mechanism involving ribosomal stress

**DOI:** 10.1038/s41598-020-59340-5

**Published:** 2020-02-13

**Authors:** Justin Zonneville, Vincent Wong, Michelle Limoge, Mikhail Nikiforov, Andrei V. Bakin

**Affiliations:** 1 Department of Cancer Genetics and Genomics, Roswell Park Comprehensive Cancer Center, Buffalo, New York 14263 USA; 2 Jacobs School of Medicine and Biomedical Sciences, University at Buffalo, Buffalo, NY 14203 USA; 3 Department of Cancer Biology, Wake Forest University, Winston-Salem, NC 27101 USA

**Keywords:** Cancer, Breast cancer, Ribosome

## Abstract

Triple-negative breast cancer (TNBC) is among the most aggressive forms of breast cancer with limited therapeutic options. TAK1 is implicated in aggressive behavior of TNBC, while means are not fully understood. Here, we report that pharmacological blockade of TAK1 signaling hampered ribosome biogenesis (RBG) by reducing expression of RBG regulators such as RRS1, while not changing expression of ribosomal core proteins. Notably, TAK1 blockade upregulated expression of p53 target genes in cell lines carrying wild type (wt) *TP53* but not in p53-mutant cells, suggesting involvement of ribosomal stress in the response. Accordingly, p53 activation by blockade of TAK1 was prevented by depletion of ribosomal protein RPL11. Further, siRNA-mediated depletion of TAK1 or RELA resulted in RPL11-dependent activation of p53 signaling. Knockdown of RRS1 was sufficient to disrupt nucleolar structures and resulted in activation of p53. TCGA data showed that TNBCs express high levels of RBG regulators, and elevated RRS1 levels correlate with unfavorable prognosis. Cytotoxicity data showed that TNBC cell lines are more sensitive to TAK1 inhibitor compared to luminal and HER2^+^ cell lines. These results show that TAK1 regulates p53 activation by controlling RBG factors, and the TAK1-ribosome axis is a potential therapeutic target in TNBC.

## Introduction

Breast cancer (BC) is the second leading cause of cancer-related death in women^1^. This heterogeneous disease includes multiple subtypes with distinct genetic, pathological and clinical features^2–4^. Among BC subtypes, triple-negative breast cancers (TNBCs, ER/PR/HER2-negative) pose special challenges due to a lack of targeted therapy and overall poor prognosis^5^, underscoring a pressing need for better therapeutic options.

Recent studies, including from our group, have implicated TGF-beta-activated kinase-1 (TAK1) in cancer progression and metastasis of breast, lung and colon cancers^6–12^. TAK1 kinase, encoded by the MAP kinase kinase kinase gene (*MAP3K7*), mediates signaling by transforming growth factor beta (TGF-β) and pro-inflammatory cytokines^13–15^. TAK1 activation involves E3-ubiquitin ligases cIAP1 and cIAP2 (cellular inhibitors of apoptosis, cIAPs), which mediate addition of K(lysine)-63-linked ubiquitin chains to RIP kinase and other signaling proteins^16^. Activated TAK1 phosphorylates Ser/Thr residues in target proteins such as IKKα/CHUK, IKKβ/IKBKB and MKK3/6, leading to stimulation of MAPK (p38, ERK, JNK) and NF-κB (nuclear factor kappa-light-chain-enhancer of activated B cells) signaling pathways^17^. TAK1 protects cells from death-inducing agents by regulating cIAPs and cFLIP, a caspase-8 inhibitor^18,19^, and may also contribute to tumor resistance to radiation and chemotherapy^20,21^. Preclinical studies with TNBC models have implicated TAK1 in tumor angiogenesis and metastases to the lungs and bone^11,22^. Evidence also supports targeting TAK1 in treating colon and pancreatic cancers^9,12^. Several pharmacological TAK1 inhibitors (TAK1-i) have been developed^23–25^. The most potent TAK1-i 5Z-7-oxozeaenol selectively inhibits TAK1 at low nanomolar amounts by irreversible inactivation of ATP-binding site^23,26^.

Advanced cancers show elevated levels of ribosome biogenesis underscoring a strategy for anti-cancer therapy targeting ribosome biosynthesis^27^. Indeed, the anti-cancer activity of various chemotherapeutic agents is associated with disruption of ribosome biosynthesis^28^. Human ribosomes comprise of two nucleoprotein subunits composed of over 80 proteins and four ribosomal RNAs. Ribosome biogenesis occurs in the nucleolus, a prominent structure within the nucleus, where ribosomal RNAs are transcribed, processed and assembled into small and larger ribosomal subunits^29^. Ribosome biogenesis requires coordinated expression of rRNA and proteins, processing of rRNA, and assembly of small 40 S and large 60 S ribosome subunits in the nucleolus, and trafficking subunits to the cytoplasm^30^. Disruption of ribosome biogenesis can cause ribosomal stress that activates tumor suppressor p53^31^. The mechanism involves accumulation in the nucleoplasm of ribosomal proteins RPL11 and RPL5, which inactivate MDM2, an E3 ubiquitin ligase mediating degradation of p53^32^. Tumor suppressor p53 is commonly mutated or deleted in TNBC and advanced cancers, indicating dysregulation of the ribosomal stress response. Better understanding mechanisms controlling ribosome biogenesis may lead to new and more effective therapies against p53-deficient breast cancers.

This study provides evidence that TAK1 signaling contributes to breast cancer by regulating expression of ribosome biogenesis factors. We showed that investigational anti-TAK1 agent down-regulated expression of ribosome biogenesis (RBG) factors, and up-regulated expression of p53-target genes in p53-wt cells. Addressing the mechanism, we found that pharmacological or molecular blockade of TAK1 signaling results in stabilization and activation of p53, and this depends on ribosomal proteins mediating the ribosomal stress response. Further, the TCGA data showed elevated ribosome biogenesis and TAK1 signaling in TNBCs compared to other breast cancer subtypes. These results suggest that TAK1 regulates ribosome biogenesis, and the TAK1-ribosome axis is a novel therapeutic target in TNBC and advanced disease.

## Results

### Inhibition of TAK1 up-regulates the p53-p21 signaling axis

**Figure 1:**
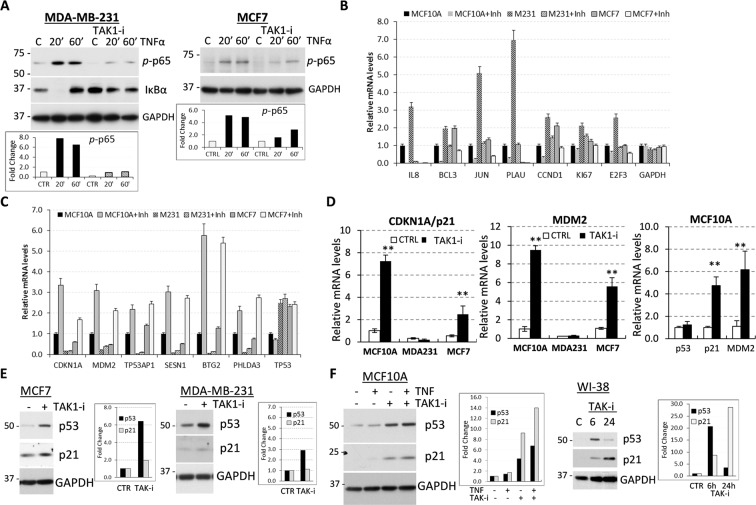
TAK1 inhibition up-regulates p53 signaling. (**A**) TAK1 inhibitor 5Z-7-oxozeaenol blocks activation of NFκB/RELA signaling. Immunoblotting of whole-cell lysates from breast cancer MDA-MB-231 and MCF7 cell lines treated with 10 ng/ml TNFα -/ + 5 µM 5Z-7-oxozeaenol. Graphs (panels **A, E, F**) show fold changes relative to control in the GAPDH-normalized band density. (**B**) Treatment with TAK1-i decreases mRNA levels of genes responsive to TAK1 signaling. Expression profiles were measured using microarrays and two independent preparations of total RNA from cells treated with TAK1-i for 6 hours. (**C**) TAK1-i increases expression of p53 target genes in MCF7 and MCF10A cell lines carrying p53 wt but not in p53-mutant MDA-MB-231. Microrray data for two independent preparations of total RNA from cells treated with TAK1-i for 6 hours. (**D**) Quantitative RT-PCR of p21 and MDM2 relative to GAPDH. Experiments were done in triplicates and repeated at least two times, **, P < 0.01. (**E**,**F**) Immunoblots of whole-cell lysates from cells treated with TAK1-i or TNFα for 24 hours, unless indicated otherwise.

TAK1 signaling in breast cancer cells contributes to tumor invasion, angiogenesis, and metastases, and TAK1 inactivation effectively blocks these responses^8,11,22,33^. To define the molecular targets of TAK1 in tumor cells, we thought to compare gene expression profiles in breast carcinoma (MDA-MB-231 and MCF7) and non-tumor MCF10A cell lines upon treatment with a selective TAK1 inhibitor 5Z-7-oxozeaenol^23^. We found that TAK1-i effectively blocked TNF-induced activation of NFκB signaling measured by phosphorylation of RELA and protein levels of a negative regulator IκB-alpha (Fig. 1A). Expression profiles were measured at 6 hours of treatment with TAK1-i in order to limit the effects associated with cell cycle arrest in G1-phase induced by 24-hour incubation (Supplementary Fig. 1). As expected, TAK1-i markedly reduced mRNA levels of known target genes for TAK1-NFκB signaling such as IL8, BCL3, JUN, PLAU, and Cyclin D1 in all tested cell lines (Fig. 1B, Supplementary Table 1). In two ER-negative cell lines (MCF10A and MDA-MB-231), TAK1-i decreased expression of about 15% of genes involved in TAK1-NFκB responses, including chemokines CXCL1, CXCL2 and IL8/CXCL8, while their levels were lower in ER-positive MCF7 cell line (Supplementary Table 1). Notably, about 15% of down-regulated genes in all three cell lines were associated with ribosomal RNA (rRNA) processing and ribosome assembly while ribosomal proteins were not regulated, suggesting involvement of TAK1 in ribosome biogenesis (Supplementary Table 1). The gene compositions in TAK1-i-induced groups were markedly dissimilar in cells carrying mutant and wt p53. Known p53 target genes^34^ constituted roughly 25% of genes commonly induced by TAK1-i in MCF7 and MCF10A cell lines carrying p53-wt, whereas these genes were not regulated by TAK1-i in MDA-MB-231 carrying mutant p53-R280K (Fig. 1C; Supplementary Table 1). TAK1 blockade induced well-known p53 targets such as p21/CDKN1A, MDM2, SESN1, and BTG2 in p53-wt cell lines without affecting p53 mRNA levels, while their basal levels were lower in p53-mutant cells (Fig. 1C). These findings were further validated by quantitative (q) RT-PCR for MDM2 and p21/CDKN1A (Fig. 1D).

Next, we assessed protein levels of p53 and p21 in cell lines with wt (MCF7, MCF10A and WI-38), and mutant p53, MDA-MB-231 (Fig. 1E,F). These cell lines exhibit p53 and TAK signaling responses^8,35^. We found that TAK1-i increased p53 protein levels in all tested cell lines, whereas up-regulation of p21 was observed only in p53-wt cells (MCF7, MCF10A, and WI-38), indicating activation of p53-p21 signaling by TAK1-i (Fig. 1E,F, and their quantification shown in bar graphs). Together these data demonstrate that TAK1 inhibition decreases TAK1-NF-κB signaling while activating p53. Notably, our data indicate that blockade of TAK1 decreases expression of ribosome biogenesis regulators.

### The contribution of signaling pathways in the TAK1-p53 link

**Figure 2:**
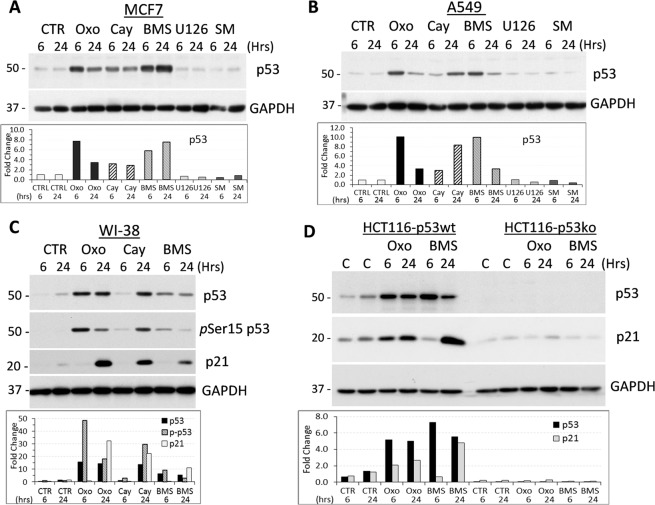
Assessment of the TAK1 signaling network in the regulation of p53. (**A-C)** Immunoblots of whole-cell lysates from MCF7, A549 and WI-38 cell lines treated for indicated time with TAK1-i 5 µM 5Z-7-oxozeaenol (Oxo) or 10 µM CAY10657 (CAY), IKKα/β inhibitor 10 µM BMS-345541 (BMS), MEK inhibitor 5 µM U0126 (U126), or Smac-mimetic cIAP inhibitor 0.1 µM TL-32711 (SM). (**D**) Human colon cancer HCT-116 and isogenic HCT-116-p53ko cell lines were treated for indicated time with TAK1-i 5 µM 5Z-7-oxozeaenol (Oxo) or IKKα/β inhibitor 10 µM BMS-345541 (BMS). Graphs show fold changes relative to control in the GAPDH-normalized band density.

TAK1 activates MAP kinases (ERK, p38) and NFκB signaling while cytokine-mediated activation of TAK1 depends on cIAP-mediated K63-ubiquitination, see above and^17^. To define a signaling mechanism leading to activation of p53 in response to TAK1 blockade, we examined responses to selective inhibitors of the aforementioned signaling pathways (see Supplementary Fig. 2) in cell line models with wild-type p53: epithelial MCF7 and A549 cell line, and mesenchymal WI-38 line. We found that increasing concentrations of TAK1-i 5Z-7-Oxozeaenol decreased phosphorylation of p65 while inducing p53 and p21 protein levels in A549 and MCF7 cell lines (Supplementary Fig. 3A). Then, we examined the response to a structurally-unrelated TAK1-IKK inhibitor, CAY10657^36^ (Supplementary Fig. 2). Treatment with TAK1-i CAY10657 increased p53 and p21 levels while reducing phospho-p65 (Supplementary Fig. 3B). Next, we evaluated the response to agents blocking the activity of TAK1 downstream signaling mediators. Treatment with a selective IKKβ inhibitor BMS-345541^37^ (Supplementary Fig. 2) induced p53 levels comparable to TAK1 inhibitors in all tested p53-wt lines (Fig. 2A–C). All three tested inhibitors of TAK1-IKK signaling reduced phosphorylation of p65 (Supplementary Figs. 3 and 4A,B). In contrast, a selective MEK inhibitor (U0126) or cIAP inhibitor/Smac-mimetic TL-32711^38^ did not induce p53 protein in MCF7 and A549 cell lines (Fig. 2A,B) although both inhibitors effectively suppressed the cognate targets (Supplementary Fig. 4C,D). In addition, we found that treatment with TNFα had no significant effect on the p53-p21 axis (Fig. 1F). Notably, induction of p53 by TAK1 blockade in MCF7 and A549 cell lines was temporal and reduced by 24 hours. This effect may associate with a negative feedback mechanism by the p53-MDM2 axis^39^. To address this possibility, we examined the response to TAK1 inhibitor alone and in combination with Nutlin 3A that disrupts the p53-MDM2 interaction^40^. We found that TAK1 blockade induced the p53-MDM2 axis at 6 hours and this response declined by 24 hours while p53, MDM2 and p21 protein levels remained high for 24 hours in cells treated with Nutlin 3A (Supplementary Fig. 5). Co-treatment with TAK1-i and Nutlin 3A resulted in stabilization of p53 and p21 protein levels at 24 hours in two tested cell lines (Supplementary Fig. 5B,C).

Then, we explored regulation of p53 signaling by TAK1-IKK inhibitors in human mesenchymal WI-38 cell line carrying wild-type p53 (Fig. 2C). Treatment with TAK1 or IKK inhibitors induced levels of p53 protein and Ser15 phosphorylation (Fig. 2C). Phosphorylation of p53 at Ser15 stimulates transcriptional activity of p53 target genes such as p21^41^. Accordingly, TAK1-IKK blockade increased p21 protein levels (Fig. 2C). To verify the role of p53 in regulation of p21 by TAK1-i, we tested the response in isogenic colon carcinoma HCT116 cell lines with p53-wt and p53-deletion. The data showed that TAK1-IKK inhibitors up-regulated p53 and p21 protein levels, while the latter response was strictly dependent on p53 (Fig. 2D). Thus, these findings showed that inhibition of TAK1-IKK signaling stabilizes p53 protein and increases expression of p53-target genes, whereas MEK-ERK signaling and activity of cIAPs are not required for this response.

### TAK1 signaling inhibitors induce nucleolar stress

**Figure 3:**
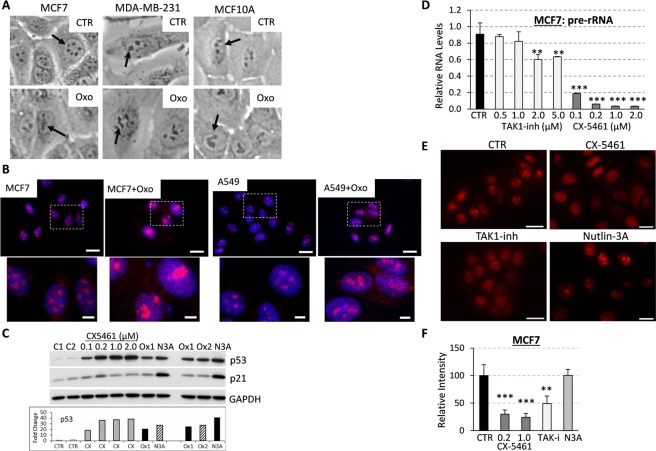
TAK1 blockade disrupts nucleolar architecture and attenuates rRNA synthesis. (**A**) Phase contrast images of the nucleus in cells treated with TAK-i 5 µM 5Z-7-oxozeaenol (Oxo) for 24 hours. (**B**) MCF7 and A549 cells were treated with 5 µM 5Z-7-oxozeaenol (Oxo) followed by staining for fibrillarin (red) and DNA (blue), bar, 20 µm. Enlarged images, bar 5 µm. (**C**) Immunoblots of whole-cell lysates from MCF7 cells treated for 24 hours with RNA pol I inhibitor CX-5461 or TAK1-i 5 µM 5Z-7-oxozeaenol (obtained from two sources Ox1, Ox2) or MDM2 inhibitor 5 µM Nutlin 3A (N3A). (**D**) Quantification of unprocessed pre-rRNA by qRT-PCR of internal transcribed spacer 1 (ITS1) in total RNA from MCF7 cells treated with CX-5461 or TAK1-i for 24 hours. Experiments were done in triplicates and repeated at least two times, **P < 0.01, ***P < 0.001. (**E**,**F**) MCF7 cells were incubated with 10 µM 5-ethynyl-uridine (EU) for 1 hour -/ + CX-5461, TAK1-i or Nutlin 3A followed by labeling with Cy3 using click-it chemistry. Images were acquired with 60X lens, bar 20 µm. (**F**) Quantification of Cy3-labeled RNA relative to control was done as described in Methods, **P < 0.01, ***P < 0.0001.

Stabilization and activation of p53 can be stimulated by various cellular stresses including DNA damage and ribosome biogenesis stress^31^. Examination of phospho-Ser139-H2AX (γH2AX), a DNA damage marker, showed no induction of DNA damage within 6 hours of TAK1-i treatment. Given down-regulation of ribosome biogenesis genes by TAK1-i, we examined whether TAK1 blockade affects the structure of nucleolus, a site of ribosome biogenesis. Disruption of the nucleolar architecture indicates an interruption in ribosome biogenesis that leads to stabilization of p53^42^. Phase microscopy revealed alterations in the nucleolar structures upon treatment with TAK1-i in both wt-p53 and mut-p53 cell lines (Fig. 3A). To validate disruption of the nucleolus, the cells were stained for fibrillarin, a nucleolar protein required for the organization and processing of rRNA^43^. Fluorescence microscopy showed that the nucleus of control-untreated cells contain 3–5 fibrillarin- stained speckles, whereas the organization of the fibrillarin speckles was altered in TAK1-i treated cells (Fig. 3B). The observed changes in the fibrillarin speckles were similar to those produced by 5-fluorouracil (5-FU) which is known to induce the nucleolar stress and ribosome biogenesis interruption by blocking rRNA processing^42^. Thus, these data suggest that inhibition of TAK1 signaling disrupts the nucleolar architecture and this may activate a p53-dependent response.

Next, we examined whether TAK1 blockade disrupts ribosomal RNA synthesis in comparison to a potent RNA polymerase I inhibitor CX5461^44^, and Nutlin 3A, a known activator of p53 that stabilizes p53 through a direct disruption of the p53-MDM2 interactions^40^. First, testing the effects on the p53-p21 axis, we found that treatment with increasing amounts of CX5461 resulted in upregulation of p53 and p21 protein levels (Fig. 3C). These effects were comparable with those of TAK1-i 5Z-7-oxozeaenol obtained from two different sources, Ox1 and Ox2 (Fig. 3C). As expected, Nutlin 3A induced robust up-regulation of the p53-p21 axis compared to CX5461 and TAK1-i (Fig. 3C). Similar results were obtained for MCF10A cells (Supplementary Fig. 6). Next, rRNA synthesis was measured by assessing levels of unprocessed pre-rRNA that contains internal transcribed spacer 1 (ITS1), which is rapidly removed following pre-rRNA synthesis and its presence indicates newly synthesized rRNA^45–47^. Quantitative RT-PCR (qRT-PCR) data revealed that TAK1-i moderately reduces pre-rRNA ITS1 levels in MCF7 cells, while CX5461 showed a more pronounced reduction in pre-RNA levels (Fig. 3D). Note, CX5461 at the concentrations ≥1 μM may induce DNA damage in some cell lines^44^.

To validate the qRT-PCR data, we evaluated *de novo* rRNA synthesis by measuring incorporation of 5-ethynyl uridine (EU) into newly synthesized RNA^48^. RNA-incorporated EU was labeled with Cy3 fluorophore using click-it chemistry^49^. Fluorescence microscopy showed the presence of bright EU-containing speckles (nucleoli) within the nucleus of control cells (Fig. 3E). As expected, CX-5461 markedly reduced the number and intensity of these speckles (Fig. 3E,F). TAK1 inhibitor also reduced the EU speckle intensities (Fig. 3E,F). Nutlin 3A did not affect the EU speckle parameters, suggesting that activation of p53 alone is not sufficient to impact rRNA synthesis. Together, these data indicate that TAK1 blockade leads to disruption of ribosome biogenesis, as indicated by changes in the nucleolus architecture and rRNA synthesis.

### Nucleolar stress and induction of p53 in response to TAK1-IKK inhibition

**Figure 4:**
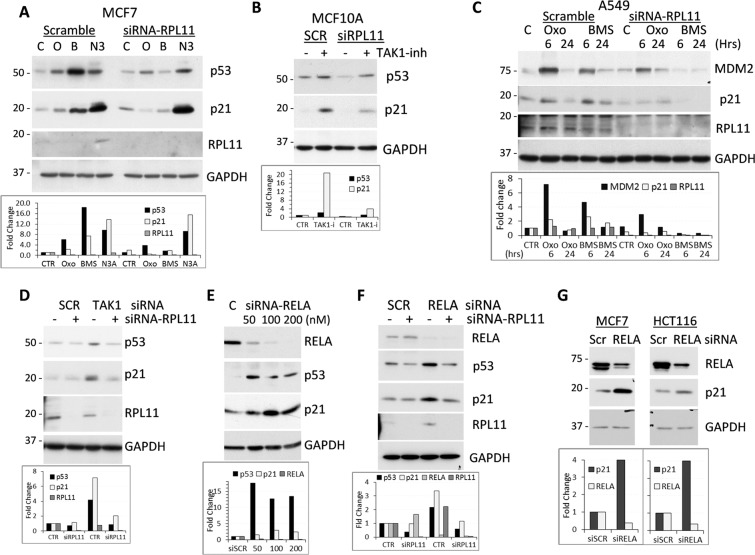
Ribosomal stress contributes to induction of p53 signaling by TAK1 blockade. (**A–C**) Cells were transfected with scrambled-control or siRNA to RPL11 followed by treatment with 5 µM 5Z-7-oxozeaenol (O or Oxo) or IKKα/β inhibitor 10 µM BMS-345541 (B or BMS). Whole-cell lysates were probed with antibodies to p53, p21 and GAPDH, a loading control. (**D–F**) A549 cells were transfected with scrambled-control or siRNA to RELA or TAK1 in combination with siRNA to RPL11. Whole-cell lysates were probed with antibodies to p53, p21 and GAPDH. (**G**) MCF7 and HCT-116 cells were transfected with scrambled-control or siRNA to RELA. Whole-cell lysates were probed with antibodies to RELA, p21, and GAPDH. Graphs show fold changes relative to control and normalized to GAPDH.

Next, we examined whether activation of p53 by TAK1 blockade is associated with disruption of ribosome biogenesis. Prior studies reveal that interruption in ribosome biogenesis leads to accumulation in the nucleoplasm of ribosomal protein RPL11 as a part of the 5 S rRNP complex, which inhibits MDM2 and stabilizes p53^35,50^. Further, depletion of RPL11 is sufficient to suppress up-regulation of p53 in response to the ribosome biogenesis (nucleolar) stress^35,50^. To assess the role of this mechanism in the TAK1-dependent activation of p53, cells were transduced with siRNA to RPL11 followed by treatment with TAK1 inhibitors. Blockade of TAK1 or IKK increased p53 and p21 protein levels in MCF7 cells transfected with scramble-control siRNA, whereas this response was greatly reduced in RPL11-depleted cells (Fig. 4A). As expected, Nutlin 3A, a MDM2 inhibitor, induced p53 and p21 proteins independently of RPL11 (Fig. 4A). Depletion of RPL11 in MCF10A or A549 cells also diminished activation of p53 by TAK1 or IKK inhibitors (Fig. 4B,C). These data implicate ribosome stress in the activation of p53 by TAK1 blockade.

To validate the results obtained with pharmacological probes, we assessed regulation of p53 in response to siRNA-mediated depletion of TAK1 and RELA. First, depletion of TAK1 in A549 cells (Supplementary Fig. 7A) resulted in up-regulation of p53 and p21 compared to scramble-control while depletion of RPL11 blocked this response (Fig. 4D). Next, we found that depletion of RELA/p65 by increasing amounts of siRNA resulted in upregulation of p53 and p21 protein levels (Fig. 4E), while depletion of RPL11 reduced siRELA-induced activation of p53 (Fig. 4F). Similar results were obtained in MCF7 and HCT116 cell lines (Fig. 4G). These findings were also verified in non-tumor MCF10A cell line (Supplementary Fig. 7B). The data show that blockade of the TAK1-RELA axis activates p53 and ribosome stress contributes to this response.

### TAK1 signaling regulates expression of ribosome biogenesis genes

**Figure 5:**
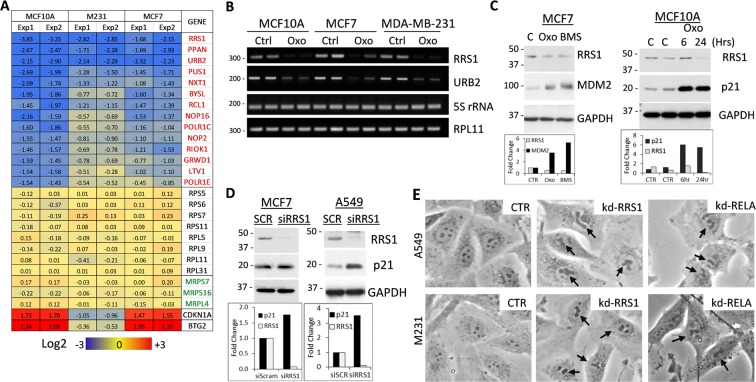
Inhibition of TAK1 signaling reduces expression of ribosome biogenesis factors (RBFs). (**A**) Changes in expression of ribosome biogenesis regulators (red gene names), ribosome core proteins (RPSs, RPLs), mitochondrial ribosome proteins (MRPSs, MRPLs) and p53 target genes CDKN1A and BTG2 in MCF10A, MDA-MB-231, and MCF7 cells treated with 5 µM 5Z-7-oxozeaenol for 6 hours. Log2 values are shown for each case. (**B**) RT-PCR analysis of RRS1, URB2, and RPL11 mRNA and control 5 S RNA in total RNA from control and 5 µM 5Z-7-oxozeaenol treated cells. (**C**) Immunoblots with whole-cell lysates from MCF7 and MCF10A cells treated with 5 µM 5Z-7-oxozeaenol (Oxo) or IKKα/β inhibitor 10 µM BMS-345541 (BMS). (**D**) MCF7 and A549 cells were transfected with scrambled-control or siRNA to RRS1 and whole-cell lysates were probed with antibodies to RRS1, p21 and GAPDH. (**E**) Phase contrast images of the nucleus in cells transfected with siRNA to RELA or RRS1 in A549 and MDA-MB-231 cells. Arrows show changes in the nucleolar structures.

To better define the mechanism of TAK1-dependent ribosomal stress, we analyzed changes in gene expression after treatment with TAK1-i for 6 hours in three cell lines described in Fig. 1. We found that mRNA levels of cytosolic and mitochondrial ribosomal proteins, including RPL11, RPS7, and MRPL4, were not affected by TAK1 blockade (Fig. 5A). In contrast, mRNA levels of ribosome biogenesis factors (RBFs) regulating rRNA processing and ribosome assembly such as RRS1, PPAN, URB2, and PUS1 were greatly reduced (Fig. 5A). In comparison, levels of p53 targets p21/CDKN1A and BTG2 were up-regulated (Fig. 5A, bottom rows). Further, RT-PCR assays confirmed down-regulation of RRS1 and URB2 by TAK1-i (Fig. 5B). In MCF7 cells, inhibitors of TAK1-i (Oxo) or IKKβ (BMS) reduced RRS1 and up-regulated MDM2 protein levels (Fig. 5C). Next, we examined whether depletion of RRS1, which controls 60 S ribosome assembly and trafficking^51,52^, affects the p53-p21 axis. Depletion of RRS1 in A549 or MCF7 cells reduced RRS1 protein levels and upregulated expression of p21 (Fig. 5D). Phase contrast microscopy showed disruption of the nucleoli in RRS1-depleted cells similar to those observed in cells after depletion of RELA (Fig. 5E). Together, these data indicate that blockade of TAK1 reduces expression of RBFs leading to disruption of ribosomal biogenesis and activation of p53.

### TAK1 signaling and ribosome biogenesis genes in breast cancer

**Figure 6:**
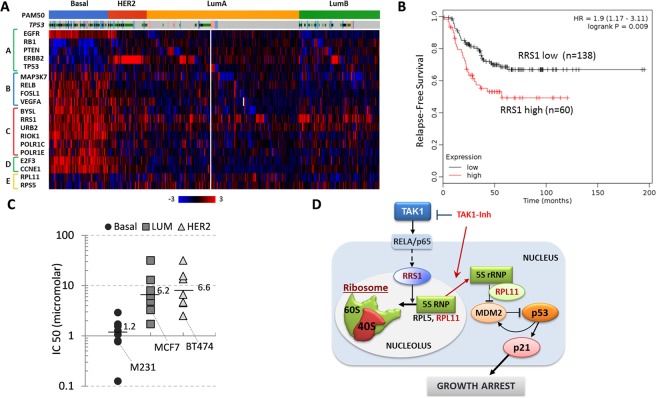
Elevated expression of ribosome biogenesis and TAK1 related genes in TNBC/basal-like cancers. (**A**) Gene expression profiles are shown in breast cancer subtypes for genes representing known breast cancer drivers (group A), TAK1 signaling (group B), ribosome biogenesis regulators (group C), cell cycle (group D), and ribosomal proteins (group E). The data were obtained using the TCGA data for 825 breast cancers ^53^. (**B**) Kaplan-Meier survival estimation of RRS1 levels and disease-free survival in TNBC patients using Breast Cancer datasets (www.kmplot.com)^55^. (**C**) Sensitivity of breast cancer cells established from basal-like/TNBC, luminal and ER-positive cancers, shown median values. The IC50 values were obtained from Genomics and Drug Sensitivity in Cancer database (^56^, Release 6.1, March 2017). (**D**) A working model of TAK1-RELA signaling in regulation of ribosome biogenesis and p53 activation.

To gain insights in clinical significance of our findings, we assessed expression of TAK1 targets and ribosome biogenesis genes in breast cancers using TCGA datasets. Analysis of the TCGA data for breast cancers^53^ revealed that basal-like breast cancers (BLBCs) express high mRNA levels of TAK1 targets (Fig. 6A, gene group B). Majority of TNBC cases exhibit BLBC molecular phenotype^2,3^ with elevated levels of EGFR receptor and deficiency in tumor suppressors p53, RB1, and PTEN (gene group A). Importantly, basal cancers express high mRNA levels of genes encoding regulators of ribosome biogenesis such as BYSL, RRS1, RIOK1, POLR1C, and POLR1E (Fig. 6A, group C). In contrast, mRNA levels of ribosomal proteins were not elevated in basal cancers (Fig. 6A, group E). Similar results were obtained using MetaBric dataset^54^ for 1,951 breast cancer patients (Supplementary Fig. 8). Evaluation of relapse-free survival (RFS) using the Kaplan-Meier plotter (www.kmplot.com)^55^ showed that high mRNA level of RRS1 is associated with poor prognosis for TNBC patients (Fig. 6B). This association was further confirmed in two TCGA datasets (Supplementary Fig. 9). Finally, we analyzed a public database for the sensitivity to TAK1-inhibitor of breast cancer cell lines^56^. The data showed that cell lines derived from TNBCs exhibited higher sensitivity (lower IC50) compared to cell lines derived from HER2 + or luminal ER-positive cancers (Fig. 6C, and Supplementary Fig. 10).

## Discussion

Accumulating evidence suggest that TAK1 is a potential therapeutic target for treatment of aggressive cancers such as metastatic breast cancer^11,22^, pancreatic cancer^9^ and colon cancer^12^. However the contribution of TAK1 to cancer remains underexplored. Here, we identified a link of TAK1-RELA signaling to the regulation of ribosome biogenesis and p53 signaling (Fig. 6D). The data suggest that blockade of the TAK1-RELA signaling axis disrupts ribosome biogenesis leading to activation of p53. In support of this conclusion, we found that blockade of TAK1-RELA signaling by pharmacological or genetic means results in up-regulation of p53 protein and expression of p53 target genes such as p21 and MDM2. The p53-p21 link was validated in isogenic p53-wt and p53-deficient HCT116 cells (Fig. 2D). Second, we found that TAK1 blockade interfered with ribosome biogenesis, affecting the nucleolar structure and reducing expression of regulators of ribosome biogenesis including RRS1 and URB2. Further, depletion of RPL11, a major mediator of p53 activation through ribosomal stress, reduced p53 activation in response to the TAK1 blockade. Third, depletion of RRS1 was sufficient to impair ribosome biogenesis and activate p53. Clinical metadata showed that TNBC/basal-like breast cancers express elevated levels of ribosome biogenesis regulators and high RRS1 levels are associated with poor prognosis. Thus, the TAK1-ribosome axis may represent a novel therapeutic target for treatment of TNBC and advance disease.

### Disruption of the TAK1-IKK-RELA signaling module leads to activation of p53

Blockade of TAK1 increased expression of p53-target genes in cells with wild-type p53 (MCF10A, MCF7, WI-38, A549 and HCT116) but not in p53-mutant (MDA-MB-231) or deficient (HCT116-p53ko) cells (Figs. 1–2). Assessment of TAK1 signaling modules revealed a major role of the TAK1-IKK-RELA axis in this response, while inhibition of MEK or cIAPs did not activate p53 (Figs. 2–3). These data are in agreement with prior reports of Verma’s group, who found elevated levels of p53 and p21 in *Ikk1/2*-deficient murine embryonic fibroblasts (MEFs)^57,58^. Consistent with our data, Gilmore’s group found inactivation of p53 in immortalized *RelA*-deficient MEFs^59^. Together, these data suggest that the TAK1-RELA axis may function through ribosome biogenesis to prevent activation of p53 in non-tumor and cancer cells.

### TAK1-p53 link and ribosome biogenesis

The inverse relationship between p53 and NFκB activities has been reported in several prior studies^57,58,60,61^ and reviewed in^62,63^. Early studies suggest that p53 and NFκB may compete for limiting pools of transcriptional transactivator p300/CBP and IKK-α-mediated phosphorylation of CBP favors a complex from p53 to NFκB^60,61^. Studies with IKK1/2 knockout fibroblasts implicated IKKβ in NFκB-mediated up-regulation of E3 ubiquitin-protein ligase MDM2 levels leading to ubiquitin-mediated degradation of p53 protein^57^. Further, IKKβ can phosphorylate p53 leading to recruitment of β-TrCP1/BTRC E3 ubuquitin ligase that ubiquitinate p53 and facilitates its degradation^58^. Besides IKKs, NFκB transcriptional target BCL3 can induce p53 degradation by up-regulating MDM2^64^, whereas p53 may repress BCL3 expression^65^. On the other hand, induction of NF-κB by oxidative stress can also stabilize and activate p53^66^. Here, we found that inhibition of TAK1 or IKKs resulted in stabilization of p53 in both wt and mutant p53 cell lines. While this observation is consistent with IKKβ-dependent mechanisms mentioned^57,58^, our data on RELA depletion suggest involvement of NFκB transcription in the regulation of p53, and this is compatible with a CBP-competing model^61^. Furthermore, our finding that depletion of ribosomal protein RPL11 reduces activation of p53 by TAK1 blockade suggests a distinct mechanism underlying activation of p53 through the ribosomal stress response. This finding was verified by pharmacological and genetic approaches in several cell lines, and separates our model from the above-mentioned mechanisms. Our data also show that p53 activation is temporal and is negatively regulated *via* a mechanism involving the p53-MDM2 interaction^39^, which is blocked by Nutlin 3A (Supplementary Fig. 5).

### Ribosome biogenesis stress mediates activation of p53 in response to blockade of TAK1

The data argue that ribosomal (nucleolar) stress mediates activation of p53 by TAK1-NFκB blockade. We found that TAK1 or RELA blockade disrupted nucleolar structures and attenuated rRNA synthesis (Figs. 3 and 5), and reduced expression of ribosome biogenesis factors (RRS1, URB2) (Fig. 5). Notably, TAK1 blockade did not alter mRNA levels of the core ribosomal proteins (e.g. RPL5, RPL11, RPS5, and RPS6) in the tested cell lines. Accordingly, depletion of RRS1 was sufficient to induce the p53-p21 axis. RRS1 is a key regulator of the 5 S RNP complex entry into the large ribosome subunit^51,67^. Interruption in ribosome biogenesis leads to accumulation in the nucleoplasm of ribosomal 5 S RNP complex containing RPL11 and other ribosomal proteins that inhibit E3-ubiquitin ligase MDM2 thereby stabilizing p53 protein^35,39,50^. Here, the data revealed that depletion of RPL11 prevented activation of p53 in response to blockade of TAK1 or RELA (Fig. 4). Together, these findings support a critical role of ribosomal stress in activation of p53 in response to TAK1 blockade. This new TAK1-ribosome axis may protect cells from abrupt interruption in ribosome production in cancer and normal cells.

The TCGA data showed elevated levels of ribosome biogenesis factors in TNBC cancers suggesting that intervention in ribosome biogenesis may selectively impede TNBCs (Fig. 6). Ribosome biogenesis consumes significant cellular energy and material resources, and defects in ribosome production may contribute to various human diseases including cancer^32^. Thus, the significance of our findings may extend beyond cancer and can be important for better understanding human diseases associated with ribosomal defects such as Treacher Collins syndrome^68^.

In summary, this work suggests that ribosome biogenesis stress mediates activation of p53 in response to TAK1 blockade. TAK1-RELA signaling may protect cells from unsolicited interruption in ribosome biogenesis. This mechanism might be particularly important for cancers exhibiting increased ribosome biogenesis such as TNBCs. In addition, this mechanism may contribute to maintenance of homeostasis by restraining activation of p53 that may lead to cell death. Thus, the TAK1-ribosome axis provides a novel strategy for therapeutic intervention in TNBC and advanced breast cancer.

## Materials and Methods

### Inhibitors, antibodies and other reagents

Inhibitors CAY10657 (Cat# 11140, CAS № 494772-86-0) and 5Z-7-oxozeaenol,Ox1, (Cat# 17459, CAS № 253863-19-3), and U0126 (Cat#70970) were obtained from Cayman Chemical (Ann Arbor, Michigan); 5Z-7-oxozeaenol, Ox2, (Cat#3604) was obtained from Tocris Bio-Techne (Minneapolis, MN); BMS-345541 (Cat#A3248), CX-5461 (Cat# A8337), Birinapant TL-32711 were from APExBio (Houston, TX); SB202190 (Cat# 559388) was from Calbiochem (EMD Millipore; Billerica, MA); Nutlin 3A (Cat# SML0580) was obtained from Sigma Aldrich (Atlanta, GA). Antibodies for: GAPDH (Cat# sc-25778), Fibrillarin (Cat# sc-25397), and IκBα (Cat# sc-371) were from Santa Cruz Biotechnology, Inc. (Santa Cruz, CA); RELA/p65 (Cat# 8242) and phospho-p65/RELA (Ser536) (Cat#3033); phospho-p53 (Ser15) (Cat# 9284) were from Cell Signaling Technology (Danvers, MA); α-Tubulin (Cat# T6074), Goat anti-Rabbit IgG (H + L)-Horseradish Peroxidase (HRP) (Cat# 170–6515) and goat anti-Mouse IgG (H + L)-HRP (CAT# 170–6516) secondary antibodies were from BIO-RAD Laboratories (Hercules, CA).

### Cell culture

Human metastatic breast carcinoma cell lines MDA-MB-231 and MCF7, human lung carcinoma cell line A549, human embryonic fibroblast cell line WI-38 were obtained from American Tissue Culture Collection (ATCC) (Manassas, VA) and cultured as recommended by ATCC. HCT116 and HCT116-p53ko was kindly provided by Dr. Bert Vogelstein (Sidney Kimmel Comprehensive Cancer Center, Johns Hopkins University). The cells were routinely screened for mycoplasma using standard procedures, and all studies made use of mycoplasma-free cells. Cell cultures were maintained in media supplemented with 10% heat-inactivated fetal bovine serum (FBS) and penicillin/streptomycin at 37 °C with 5–10% CO_2_ in a humidified incubator.

### Immunoblot analysis

A detailed description of immunoblotting has been reported elsewhere^10,11^. Briefly, whole-cell lysates were collected using NP40 Lysis Buffer (0.88% NP-40, 132 mM NaCl, 44 mM Hepes, 8.8 mM NaF) supplemented with 2 mM sodium orthovanadate, 1 mM PMSF and 1X Protease Inhibitor Cocktail (Cat# 11836153001; Roche; Basel, Switzerland). Prior to lysis, cells were grown to 70–80% confluency and, if necessary, treated with 10 ng/mL TNFα. Inhibitors were added 1 hour prior to cytokine treatment. Protein concentrations were measured using the Bio-Rad DC Protein Assay according to the manufacturer’s instructions. Proteins were resolved using SDS-PAGE and transferred to nitrocellulose membranes (Cat# 162–0112; BIO-RAD). Transfer was validated by Ponceau S staining. Protein bands were visualized using ECL chemiluminescent reagent (Cat# 32209; Pierce). Relative changes in protein levels were quantified using ImageJ software version 1.52a and presented as fold change in the GAPDH-normalized band density relative to control.

### q-RT-PCR

Procedures were performed as described in^69^. Briefly, cells were seeded in media containing 10% serum which was changed to 5% serum or serum-free media the following day. RNA extraction was performed using the TRIzol Reagent (Cat# 15596-026; Invitrogen) according to the manufacturer’s instructions. cDNA samples were prepared from equal amounts of RNA using M-MLV RT (Cat# M1701; Promega; Madison, WI), and then amplified using 5X HOT FIREPol EvaGreen qPCR Mix Plus (ROX) (Cat# 08-24-00001; Solis Biodyne, Tartu, Estonia) in the Applied Biosystems StepOnePlus Real-Time PCR System (Thermo Fisher Scientific; Waltham, MA). Samples were run in triplicate. Results were analyzed as follows: threshold cycle (Ct) values were normalized using the mean Ct for the reference gene, 5 SrRNA, defined as ΔCt = Ct (test gene) – Ct (mean for the reference gene). The final data were presented as the fold change (FC) between the test and control samples, defined as FC = 2^-(ΔCt (test gene) – ΔCt (mean for control)). Human primer sequences are as follows: CDKN1A/p21, (Forward: TTAGCAGCGGAACAAGGAGT, Reverse: GCCGAGAGAAAACAGTCCAG); MDM2, (Forward: CAGCTTCGGAACAAGAGACC, Reverse: GCAGTTACGCCAGAGGTAGC); p53/TP53, (Forward: CCCCTCCTGGCCCCTGTCATCTTC, Reverse: GCAGCGCCTCACAACCTCCGTCAT); GAPDH (Forward: GGATTTGGTCGTATTGGGC, Reverse: GGAAGATGGTGATGGGATT); and 5S rRNA (Forward: GGCCATACCACCCTGAACGC, Reverse: AGCCTACAGCACCCGGTATT); ITS1/pre-rRNA (Forward: CCGCGCTCTACCTTACCTACCT, Reverse: GCATGGCTTAATCTTTGAGACAAG).

### RT-PCR

Total RNA was prepared as for q-RT-PCR and RT-PCR reaction were performed as described in^22^. Human primer sequences are as follows: RRS1, (Forward: GGCATCCGTCCCAAGAAGAA, Reverse: TACTCTGGTGTCCGGTAGGG); URB2, (Forward: AGCAGTTGGAAAGCATCCTG, Reverse: CCCCTTTTGCAAGTAACCAA); RPL11, (Forward: ACAGACTGACGCGAGCA, Reverse: AGGAACACATCGATCTGGGTA); CDKN1A/p21, (Forward: TTAGCAGCGGAACAAGGAGT, Reverse: GCCGAGAGAAAACAGTCCAG); MDM2, (Forward: CAGCTTCGGAACAAGAGACC, Reverse: GCAGTTACGCCAGAGGTAGC); p53/TP53, (Forward: CCCCTCCTGGCCCCTGTCATCTTC, Reverse: GCAGCGCCTCACAACCTCCGTCAT); and 5SrRNA (Forward: GGCCATACCACCCTGAACGC, Reverse: AGCCTACAGCACCCGGTATT).

### Flow cytometry

For cell cycle analysis, cells were seeded at 750,000 in 10 cm^2^ dishes and left to attach overnight. Cells were treated with 5 µM of 5Z-7-Oxozeaenol for 6 and 24 hours. Collected cells were fixed in ice cold 70% ethanol for 1 hour and stained with Krishan DNA Buffer for 2 hours at RT. Krishan DNA Buffer consisted of the following reagents: Propidium Iodide, Sodium Citrate, RNAase A, NP40, and HCl. Samples were sorted using a BD LSRFortessa cytometer running Diva version 6.1.1 and cell cycle analysis was done using Modfit LT software version 4.1.7. Cell cycle analysis experiments were repeated three times with representative histograms shown.

### Microscopy

Microscopy experiments were done as described in^69^. Briefly, cells were grown on glass coverslips (22 × 22 mm) and treated with inhibitors for 24 hours, then fixed with 4% PFA and permeabilized with 0.05% Triton X-100. For fibrillarin staining samples were blocked with 3% milk in PBS for 30 min at room temperature. The cells were incubated for 1 h with antibodies to fibrillarin (1:400) in 1% milk/PBS followed by incubation for 30 min with Texas red–conjugated secondary antibody (1:500) at room temperature. Fluorescence images were taken with a Plan Apochromat 60×/1.40 NA oil objective at ambient temperature using Nikon TE2000-E inverted microscope equipped with a charge-coupled device camera (CoolSNAP HQ; Photometrics). The images were acquired using MetaVue imaging software (v7.7.3, Molecular Devices).

### rRNA synthesis

rRNA synthesis was evaluated by pulse-labeling of *de novo* synthesized RNA with 5-ethynyl uridine followed by coupling of fluorophore using click-it chemistry. Briefly, cells were grown on glass cover slips (22 × 22 mm) and treated with inhibitors for 6 hours, followed by a 1 hour pulse with 5-ethynyl uridine (Cat# PY7563, Berry & Associates, Dexter, MI). Cells were fixed in 4% PFA and permeabilized with 0.05% Triton X-100. To label RNA with Cy3 fluorophore, cells were subjected to ‘click-it’ reaction with Cu(II)SO_4_, Tris-pH 8.5, Cy3-azide, THTPA, and ascorbic acid for 1 hour, followed by wash and staining with Hoechst. Cover slips were mounted onto glass-slides and fluorescence images were acquired with a 60× oil lens using Nikon TE2000-E microscope as described above. Fluorescence intensity of the nucleus was evaluated for >30 cells/case from at least 3 randomized fields in each case by using MetaVue imaging software. Experiments were repeated at least 2 times.

### Metadata analysis

The Kaplan–Meier curves of recurrence-free survival in BC patients were generated using Kaplan-Meier Plotter^55^ (http://kmplot.com/analysis/index.php?p=service). Metastasis-free survival data were obtained using a published dataset of 295 BC patients^70^ and a prognostic *PROGgeneV2* tool^71^, http://genomics.jefferson.edu/proggene/. Heat-map of gene expression profiles was generated with the TCGA BC dataset using the cBioPortal for Cancer Genomics online tool https://www.cbioportal.org/.

### Statistical analysis

Data in each experiment was compared using the Student’s *t* test. Statistical significance was achieved when *P* < 0.05.

## Supplementary infromation

Supplementary information.
Supplementary Figures.
