# Supplementary material for: TAK1 signaling regulates p53 through a mechanism involving ribosomal stress
Source: Sci Rep. 2020 Feb 13;10:2517. doi: 10.1038/s41598-020-59340-5 (PMC7018718; doi:10.1038/s41598-020-59340-5)
Supplement: Supplementary file 2 — Supplementary Figures. [file 41598_2020_59340_MOESM2_ESM.pdf]

Supplementary Figure 1

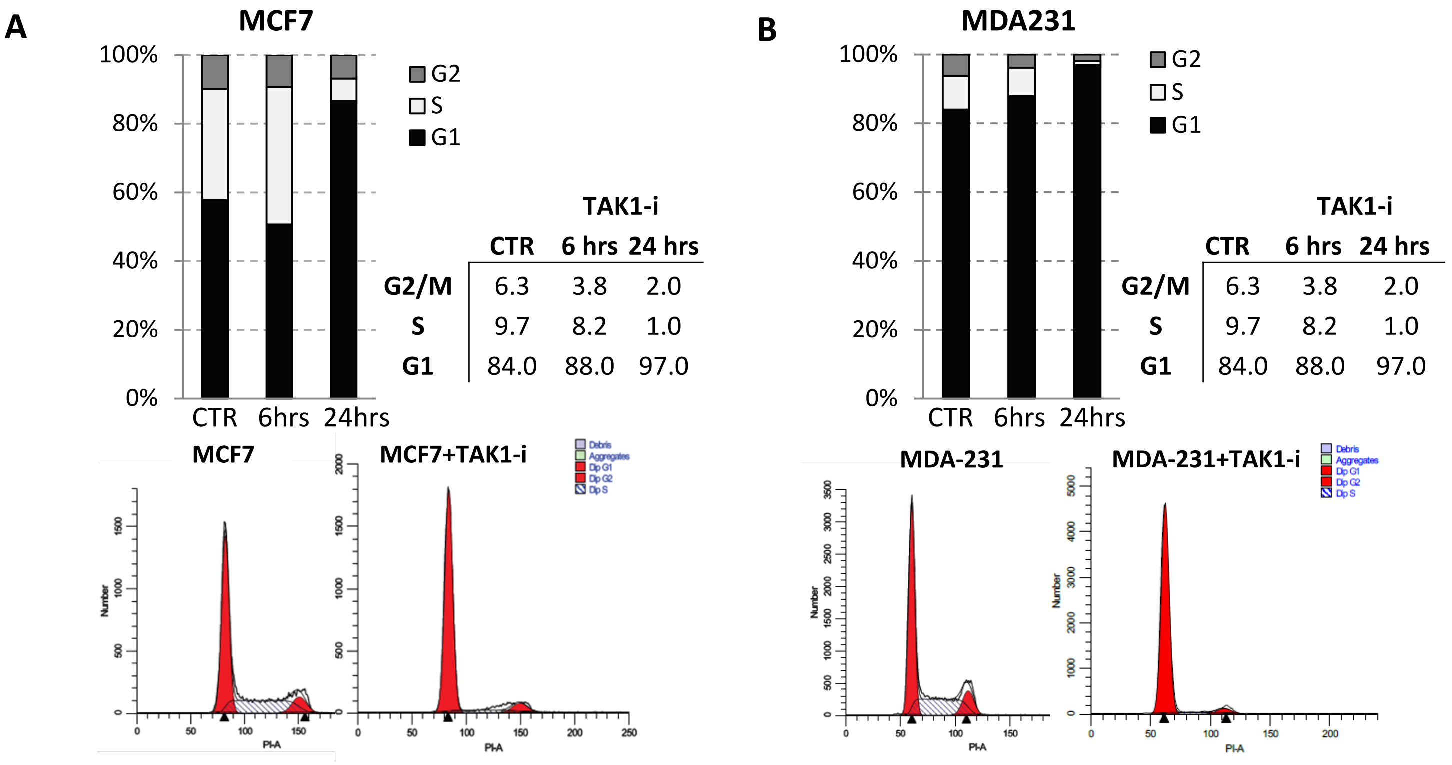

Suppl. Fig. 1. TAK1 inhibitor induces G1-arrest. (**A-B**) Breast cancer MDA-MB-231 and MCF7 cell lines were treated with 5μM 5Z-7-oxozeaenol, TAK1 inhibitor, for indicated time. Cells were stained with propidium iodide and subjected to cell cycle analysis using flow cytometry.

Supplementary Figure 2

TAK1 inhibitor (Oxo)

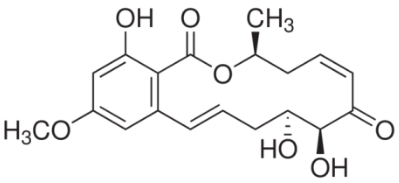

5Z-7-oxozeanol, IC50=8nM

TAK1 inhibitor (CAY)

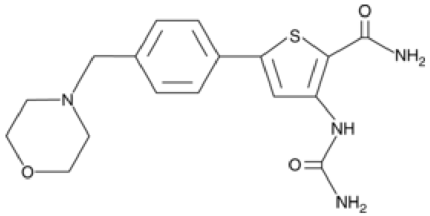

CAY10657 (CAS 494772-86-0)
IC50=50nM

IKK-β inhibitor (BMS)

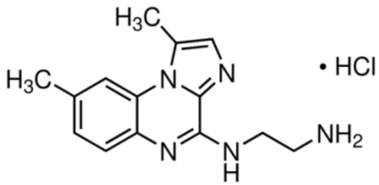

BMS-345541, IC50=200nM

MEK inhibitor (U0126)

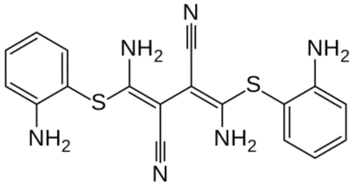

IC50= 60nM

CIAP blocker; Birinapant, smac-mimetic (SM)

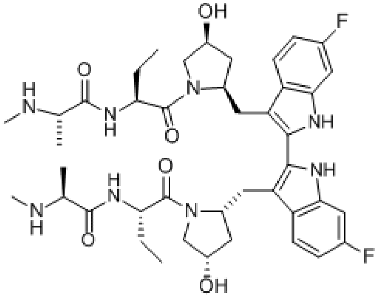

TL-32711, inhibits targets at <45nM

RNA pol I inhibitor (CX5461)

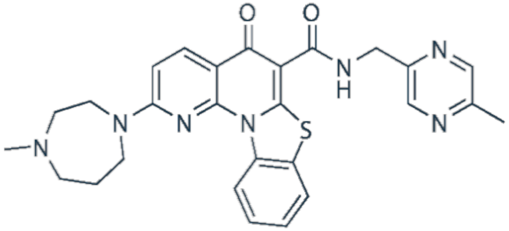

CX-5461, IC50=142nM

Suppl. Fig. 2. Structures of inhibitors utilized in the study.

Supplementary Figure 3

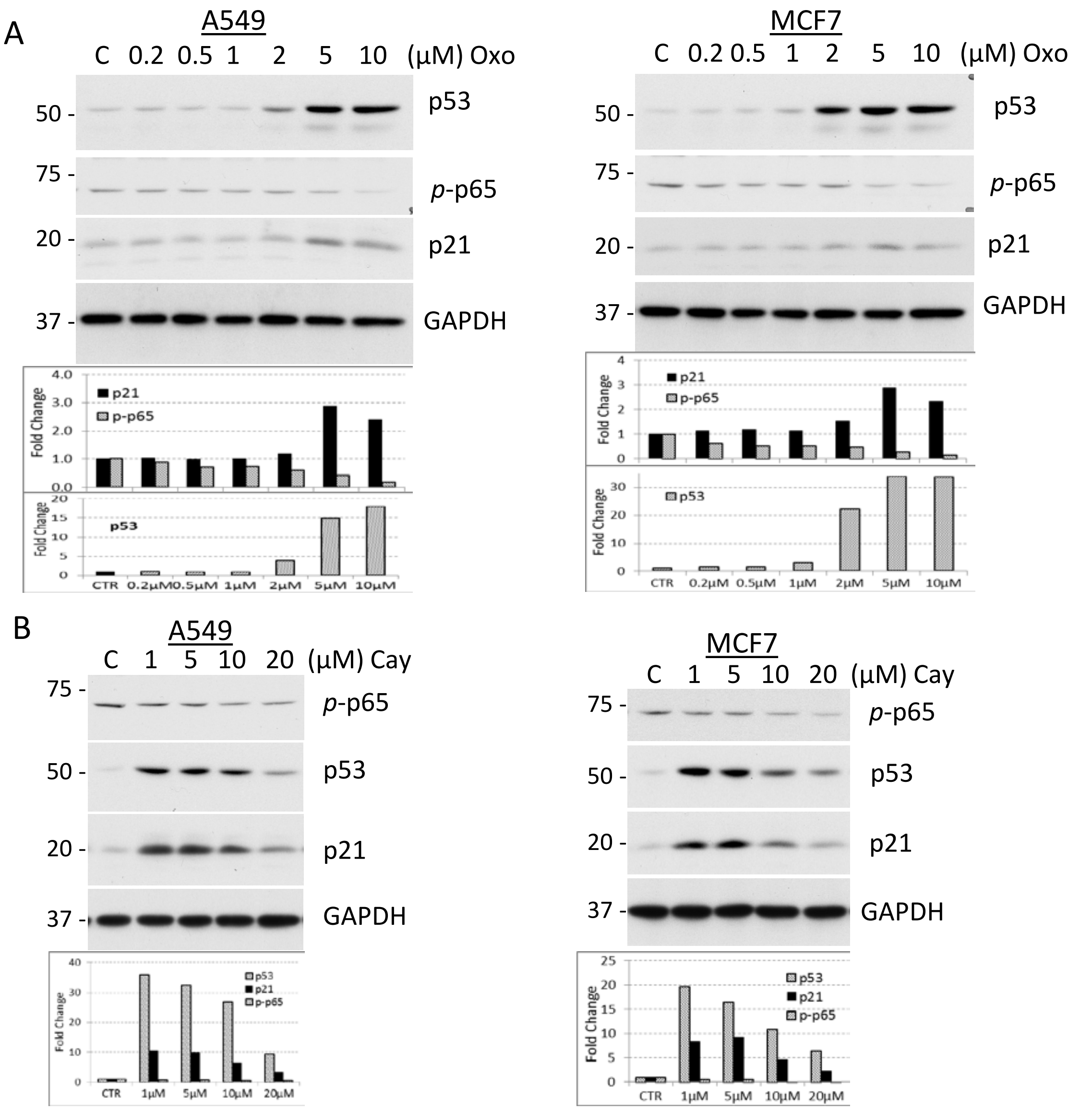

Suppl. Fig. 3. Evaluation of the response to TAK1 inhibitors in p53-wt cell lines. Lung cancer A549 and breast cancer MCF7 cell lines were treated with vehicle control (DMSO) or increasing amounts of TAK1 inhibitors, 5Z-7-oxozeaenol for 6 hours (A) or CAY10657 for 24 hours (B). Whole-cell lysates were probed with antibodies as indicated.

Supplementary Figure 4

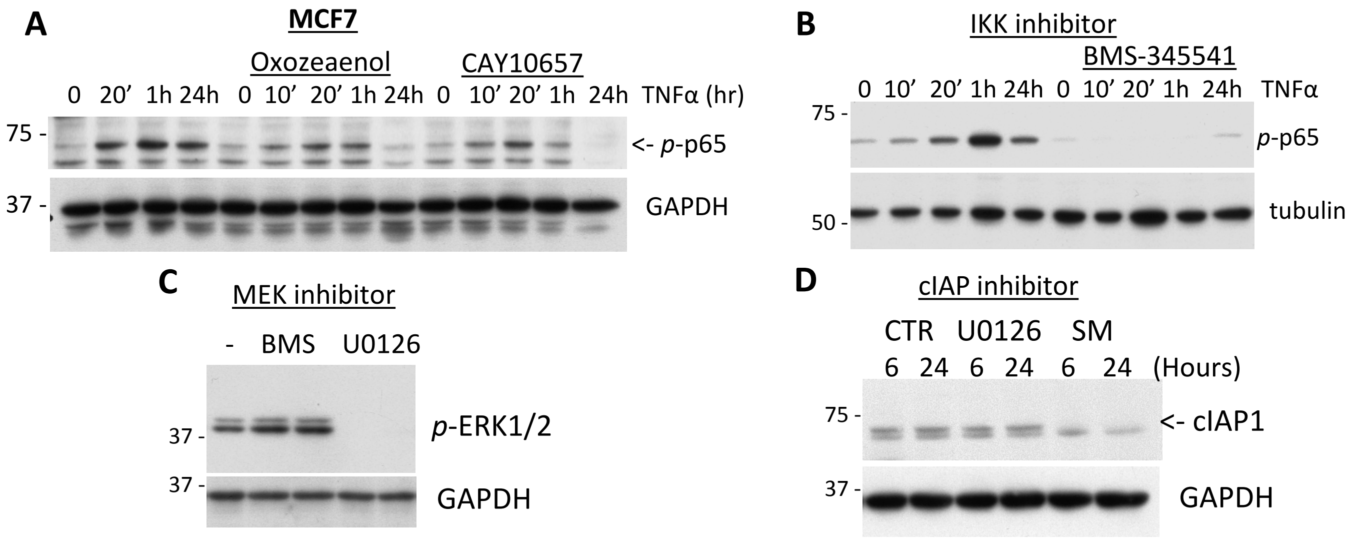

Suppl. Fig. 4. Evaluation of inhibitors in cancer cells. (**A-B**) Breast cancer MCF7 cells were treated with 10ng/ml TNFα -/+TAK1 inhibitors 5µM 5Z-7-oxozeaenol or 10µM CAY10657, or IKK inhibitor 10µM BMS-345541 for indicated time. (**C-D**) Breast cancer MDA-MB-231 cells were treated with MEK inhibitor 5µM 0126, IKK inhibitor 10µM BMS-345541, or Smac-mimetic 100nM Birinapant. Cells were probed with antibodies to phospho-p65/RELA, cIAP1/BIRC2, or phospho-ERK1/2 and GAPDH or tubulin as loading controls.

Supplementary Figure 5

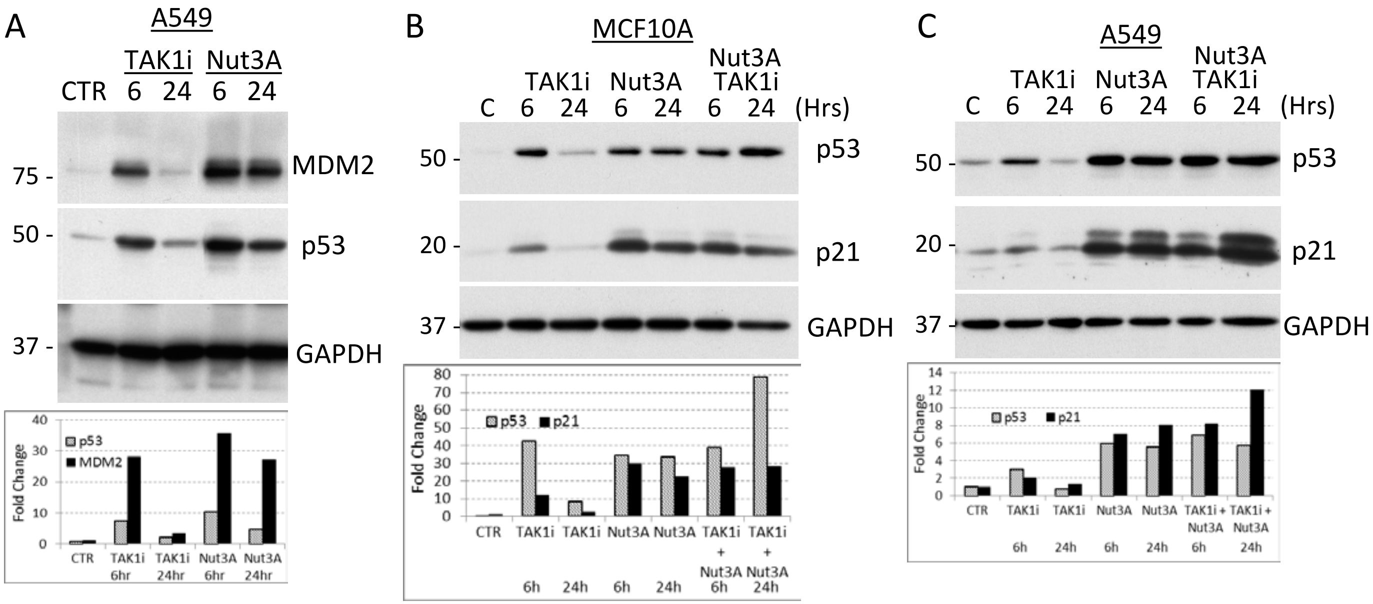

Suppl. Fig. 5. Immunoblotting of whole-cell lysates from A549 and MCF10A cell lines treated with DMSO (control), TAK1 inhibitor 5µM 5Z-7-oxozeaenol or 5µM Nutlin-3A for 6 and 24 hours alone or in combination.

Supplementary Figure 6

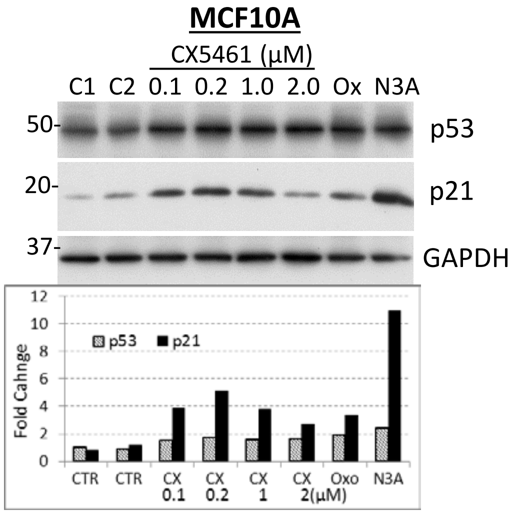

Suppl. Fig. 6. Evaluation of p53-p21 signaling in response to RNA Pol I inhibitor, TAK1 inhibitor and Nutlin3A. Non-tumor MCF10A cells were treated with 0.1-2.0µM RNA Pol I inhibitor, 5µM TAK1 inhibitors 5Z-7-oxozeaenol or 5µM MDM2 inhibitor Nutlin-3A for 6 hours.

Supplementary Figure 7

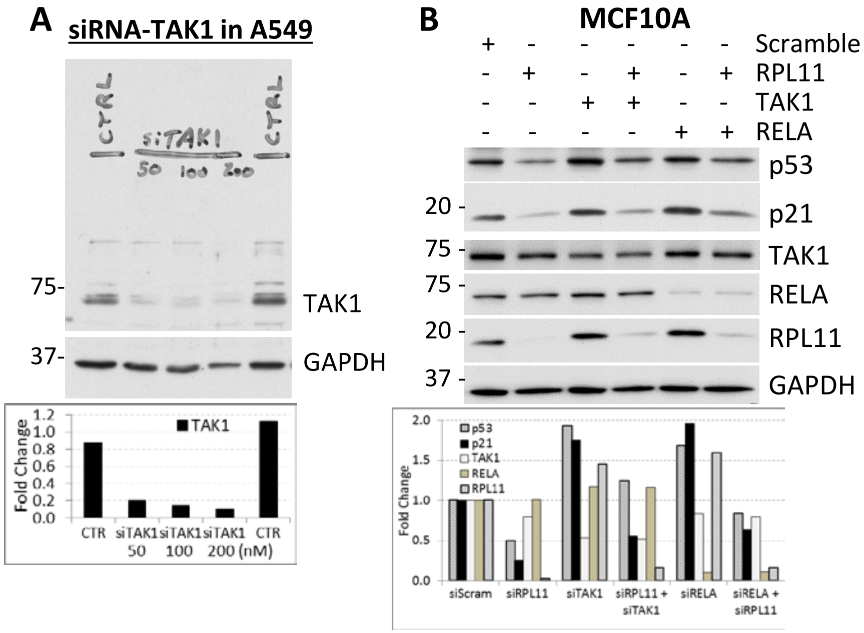

Suppl. Fig. 7. Knockdown of TAK1 and RELA using siRNA. (**A**) A549 cells were transfected with scrambled-control or siRNA to TAK1 (50-200nM) and probed with antibody to TAK1 and GAPDH, loading control. (**B**) MCF10A cells were transfected with scrambled-control or siRNA to RPL11 in combination with siRNA to RELA or TAK1. Whole-cell lysates were probed with antibodies to p53, p21, TAK1, RELA, and GAPDH.

Supplementary Figure 8

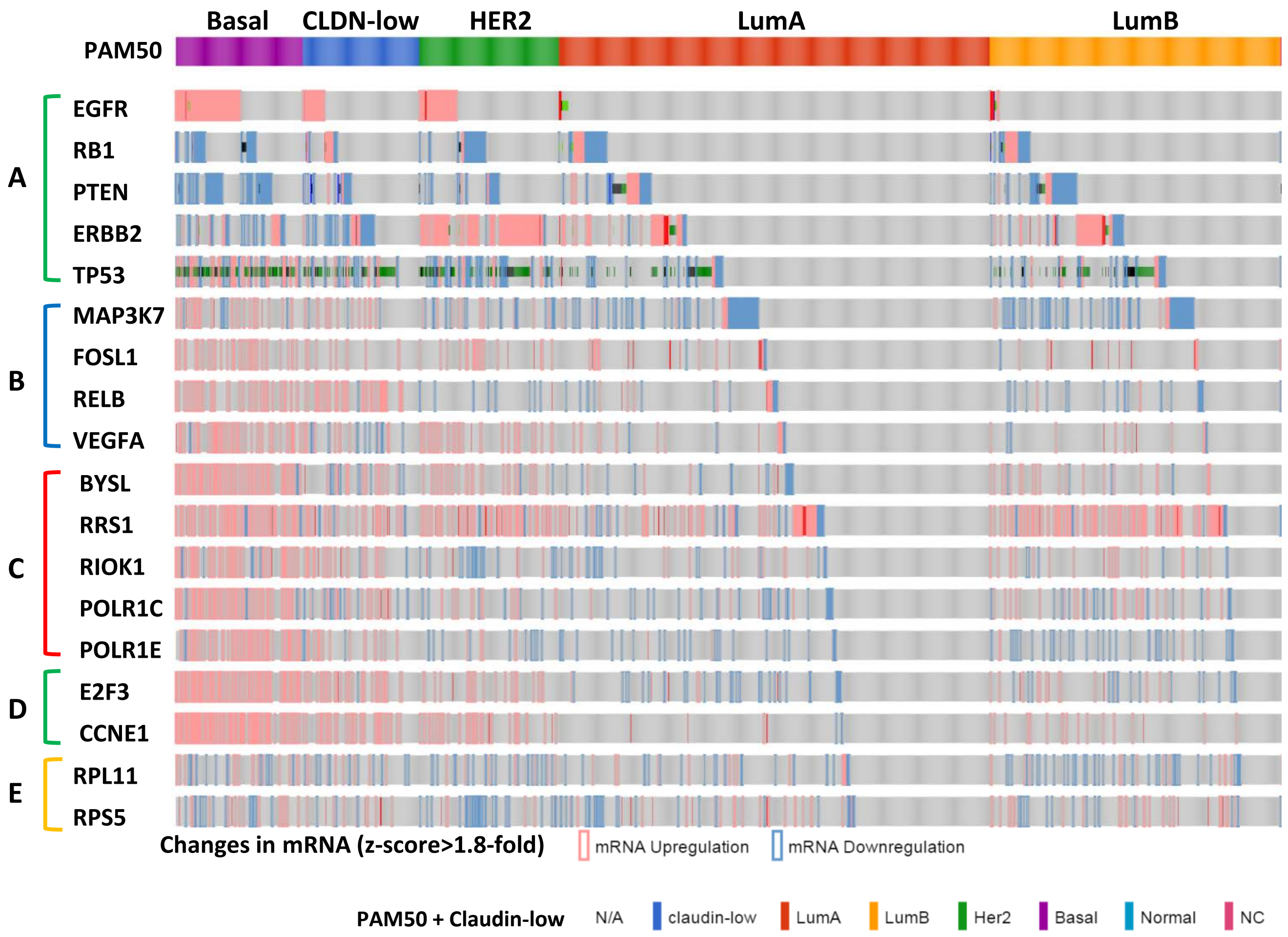

Suppl. Fig. 8. Elevated expression of ribosome biogenesis and TAK1 related genes in TNBC/basal-like cancers. (**A**) Gene expression profiles are shown in breast cancer subtypes for genes representing known breast cancer drivers (group A), TAK1 signaling (group B), ribosome biogenesis regulators (group C), cell cycle (group D), and core ribosomal proteins (group E). The data were obtained using the TCGA MetaBric dataset (Pereira et al., 2016).

Supplementary Figure 9

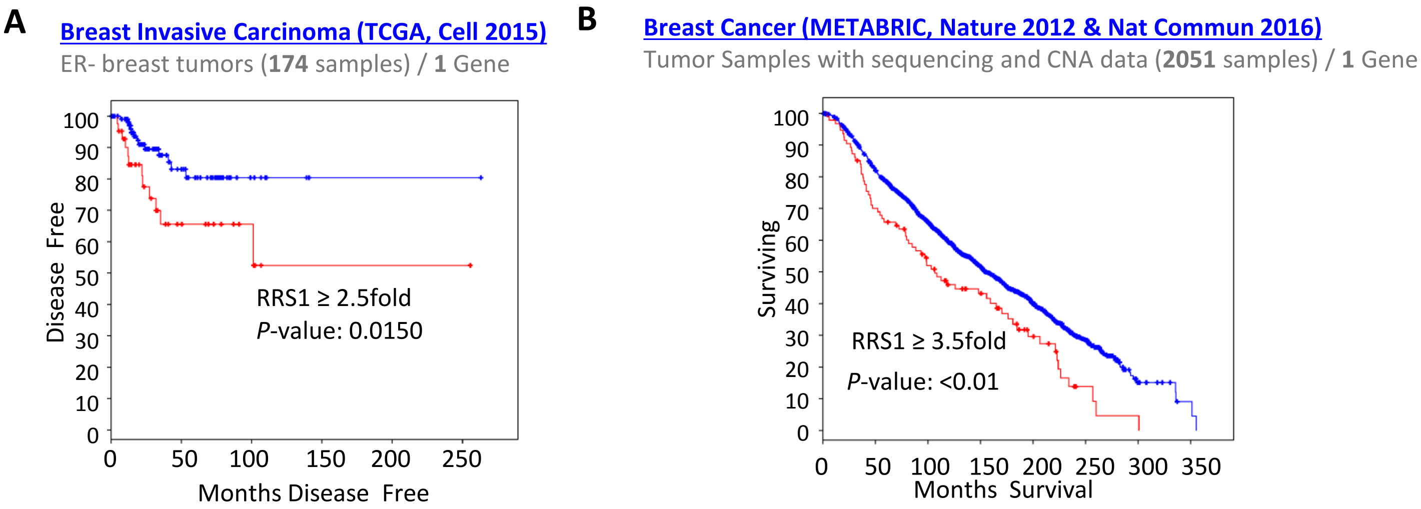

Suppl. Fig. 9. Kaplan-Meier survival estimation of RRS1 levels and disease-free survival in breast cancer patients using the Breast Cancer TCGA datasets: (**A**) ER-negative (TCGA, Cell 2015); (**B**) the MetaBric dataset (Pereira et al., 2016).

Supplementary Figure 10

IC50 values (µM) for 5Z-7-oxozeaenol

Cell Line	Basal	LUM	HER2
DU-4475	0.125		
CAL-51	0.769		
MDA-MB-231	0.796		
CAL-120	1.06		
MDA-MB-468	1.26		
BT-549	1.56		
MDA-MB-436	1.71		
Hs-578-T	2.9		
JIMT-1		1.72	
MCF7		3.28	
HCC70		4.84	
BT-483		6.24	
T47D		7.99	
HCC1428		12.9	
ZR-75-30		31.6	
BT-474			2.49
HCC1954			4.51
MDA-MB-361			4.65
MDA-MB-453			6.59
HCC2218			13.5
HCC202			15.5
ZR-75-30			31.6

The data were obtained from public database: Genomics of Drug Sensitivity in Cancer (GDSC): a resource for therapeutic biomarker discovery in cancer cells (Nucl. Acids Res. 2013; PMID:23180760); **Release 6.1 (Mar 2017)**
